# Arterial myocardial revascularization with right internal thoracic artery and epigastric artery in a patient with Leriche’s syndrome

**DOI:** 10.1186/1749-8090-8-53

**Published:** 2013-03-23

**Authors:** Dmitry Bobylev, Felix Fleissner, Ruoyu Zhang, Axel Haverich, Issam Ismail

**Affiliations:** 1 Department of Cardiothoracic, Transplantation and Vascular Surgery, Hannover Medical School, Carl-Neuberg Str. 1, Hannover, 30625, Germany; 2 Institute for Molecular and Translational Therapeutic Strategies (IMTTS), Hannover Medical School, Carl-Neuberg Str. 1, Hannover, 30625, Germany

**Keywords:** Total arterial revascularization, Right internal thoracic artery, Epigastric artery, Leriche’s syndrome

## Abstract

Concomitant coronary artery disease (CAD) and Leriche’s syndrome is clinical scenario which poses a challenge to cardiovascular surgeons. This report describes a case of arterial myocardial revascularization in a patient with CAD and Leriche’s syndrome by means of right internal thoracic artery harvested with right epigastric artery in situ fashion, performed in addition to simultaneous aorto-bifemoral bypass.

## Background

Leriche’s syndrome in patients with severe coronary artery disease is difficult to surgical manage. In these patients, the internal thoracic-epigastric arteries are critical collateral pathways used to compensate for the occlusion of the infrarenal aorta and bilateral iliac arteries that play a role in the choice of operating strategy. Here, a technique is described which provides the benefits of complete arterial revascularization with right internal thoracic artery (RITA) and right epigastric artery (EA) at the time of simultaneous coronary artery bypass grafting (CABG) and aorto-bifemoral bypass surgery.

## Case presentation

**Figure 1:**
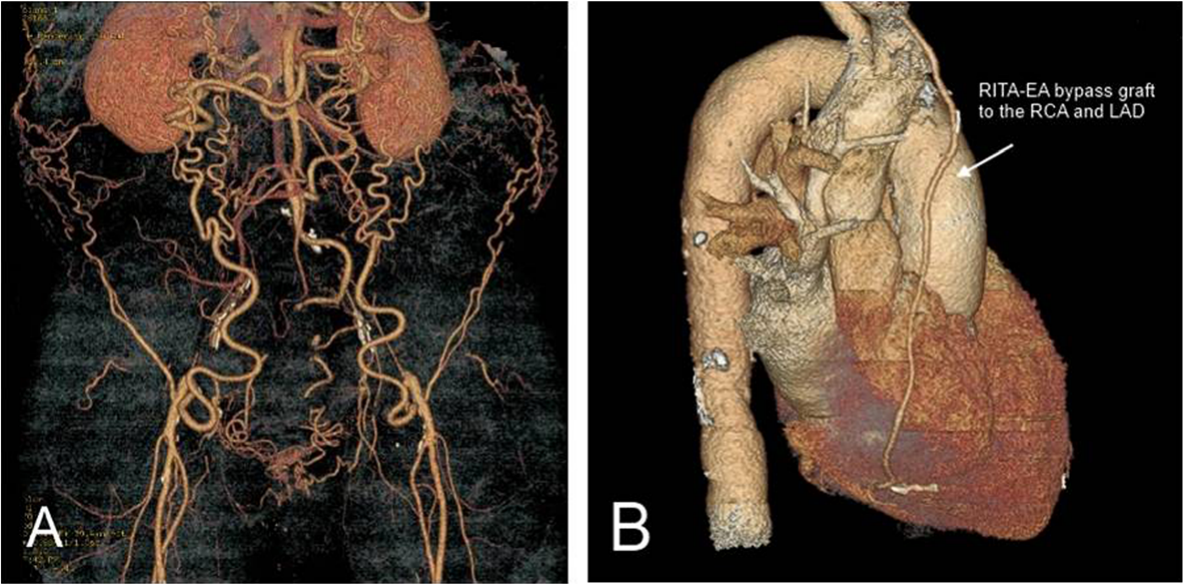
**The preoperative and postoperative 3D reconstruction from CT scan.A**) A computed tomography image showing total occlusion of the distal portion of the aorta with collaterals to the lower extremities, **B**) A postoperative cardiac computed tomography demonstrated patency of the sequential RITA – IEA bypass graft to the RCA and proximal LAD.

A 69-year-old male was admitted due to progressive multifocal atherosclerosis with resting pain in the lower limbs and angina pectoris. A CT scan revealed occlusion of the infrarenal aorta und bilateral common iliac arteries, indicating Leriche’s syndrome. Collateral circulation was established through the internal thoracic arteries, the epigastric arteries and some lumbar arteries (Figure 1A). A Coronary angiogram demonstrated significant stenoses in the proximal left anterior descending artery (LAD) and in the middle right coronary artery (RCA). Due to these findings, after discussion with cardiologists, the option of simultaneous arterial myocardial revascularization and aorto-bifemoral bypass was chosen.

After complete median sternotomy and median laparotomy, the RITA and right EA were harvested, as there was sufficient *in situ* length. Following the establishment of cardiopulmonary bypass and aortic cross-clamping, myocardial revascularisation was performed with side-to-side anastomosis of the RITA-EA graft to RCA and end-to-side of the proximal LAD. Subsequently, during reperfusion, the patient underwent aorto-bifemoral bypass using a 16-8 mm Y-polytetrafluoroethylene graft.

The postoperative course was uneventful. The peripheral pulse on the lower limbs was apparently palpable. The patient was discharged home on the postoperative day 10 in good physical and psychological condition. A postoperative CT scan demonstrated patency of the sequential arterial bypass (Figure 1B). In a telephone follow-up 6 months after surgery with patient reported about no event of angina pectoris.

Patients with coronary artery disease (CAD) and aortoiliac occlusive disease are a high-risk subgroup of patients. In these patients, the internal thoracic artery (ITA), as the preferred conduit for CABG in most coronary operations, may be essential for collateral blood supply in cases of obstruction of the aortoiliac arterial system. The use of the ITA for CABG in these situations may cause significant ischaemia in the lower extremity [1]. On the other hand, aortic cross-clamping before coronary surgery may have a deleterious effect on cardiac function, ultimately leading to myocardial infarction [2,3]. The key to the management of such patients is to select the appropriate strategy based on the perceived risk of potential ischaemia of the lower extremities, as well as a detailed knowledge of the anatomy of collateral arterial circulation. One-stage surgery, i.e., myocardial revascularization and simultaneous vascular bypass operation, has been proposed in this situation [4].

In a patients with aortic or an iliac occlusion, collateral pathways develop as a blood supply route to the lower extremities. The IMA-EA pathway is known to be one of the major collateral routes, but has rarely been used in coronary revascularization [5]. The EA in the patient presented here appeared normal with no gross hypertrophy or calcification, except for its large calibre due to the collateralization. Moreover, harvesting of EA was performed to run concomitantly with laparotomy. Other collateral pathways, such as the inferior mesenteric and lumbar arteries, were also developed, enabling the maintenance of blood supply during the operation. By using the RITA-EA graft we were able to leave the left internal thoracic artery (LITA) in situ. Since the collateral flow to the lower body was in part established through the LITA, we left this important collateral untouched. If we would have used in situ bilateral IMA grafts for LAD and RCA, the established collaterals would have been harmed.

The *in situ* RITA - EA graft allowed us to reach the right coronary artery, as well as the proximal left anterior descending artery. We did not use Saphenous vein, because of our concern of wound infection after graft harvesting in Leriche’s patients. Moreover, the long-term patency of arterial grafts has been clearly demonstrated to be superior to that of vein grafts.

## Conclusion

It is believed there is a minor role for combining CABG with aorto-bifemoral repair, except for in rare patients with symptomatic CAD and ischaemia of lower extremities. However, it is also thought that our experience with this technique can be useful in decision-making in patients with concomitant Leriche’s syndrome with the IMA-EA collateral pathway as a major route to the lower extremities.

## Consent

Written informed consent was obtained from the patient for publication of this case report and accompanying images. A copy of the written consent is available for review by the Editor-in-Chief of this journal.

## Competing interests

The authors declare that they have no competing interests.

## Authors’ contributions

All authors have no financial or other interests regarding the submitted manuscript. DB conceived the study, provided the information of the patient, performed literature search, wrote and reviewed the manuscript, FF participated in drafting the manuscript, RZ participated in the coordination of this study, AH participated in drafting the manuscript, supervised and reviewed the manuscript, II was the operating surgeon of the patient, supervised and reviewed the manuscript. All authors read and approved the final manuscript.
